# Quantifying the Individual and Combined Effects of Short-Term Heat Stress at Booting and Flowering Stages on Nonstructural Carbohydrates Remobilization in Rice

**DOI:** 10.3390/plants13060810

**Published:** 2024-03-12

**Authors:** Aqib Mahmood, Wei Wang, Muhammad Ali Raza, Iftikhar Ali, Bing Liu, Leilei Liu, Yan Zhu, Liang Tang, Weixing Cao

**Affiliations:** 1 National Engineering and Technology Center for Information Agriculture, Key Laboratory for Crop System Analysis and Decision Making, Ministry of Agriculture and Rural Affairs, Engineering Research Center for Smart Agriculture, Ministry of Education, Jiangsu Key Laboratory for Information Agriculture, Jiangsu Collaborative Innovation Center for Modern Crop Production, Nanjing Agricultural University, Nanjing 210095, China; aqib.mahmood@iub.edu.pk (A.M.); 2018201094@njau.edu.cn (W.W.); dr.iftikharali@uaf.edu.pk (I.A.); bingliu@njau.edu.cn (B.L.); yanzhu@njau.edu.cn (Y.Z.); 2 National Research Centre of Intercropping, The Islamia University of Bahawalpur, Bahawalpur 63100, Pakistan; 3 Department of Agronomy, University of Agriculture, Faisalabad 38000, Pakistan

**Keywords:** rice (*Oryza sativa* L.), nonstructural carbohydrates, translocation, heat tolerance

## Abstract

Rice production is threatened by climate change, particularly heat stress (HS). Nonstructural carbohydrates (NSCs) remobilization is a key physiological mechanism that allows rice plants to cope with HS. To investigate the impact of short-term HS on the remobilization of nonstructural carbohydrates (NSCs) in rice, two cultivars (Huaidao-5 and Wuyunjing-24) were subjected to varying temperature regimes: 32/22/27 °C as the control treatment, alongside 40/30/35 °C and 44/34/39 °C, for durations of 2 and 4 days during the booting, flowering, and combined stages (booting + flowering) within phytotrons across the years 2016 and 2017. The findings revealed that the stem’s NSC concentration increased, while the panicle’s NSCs concentration, the efficiency of NSCs translocation from the stem, and the stem NSC contribution to grain yield exhibited a consistent decline. Additionally, sugar and starch concentrations increased in leaves and stems during late grain filling and maturity stages, while in panicles, the starch concentration decreased and sugar concentration increased. The heat-tolerant cultivar, Wuyunjing-24, exhibited higher panicle NSC accumulation under HS than the heat-sensitive cultivar, Huaidao-5, which had more stem NSC accumulation. The flowering stage was the most vulnerable to HS, followed by the combined and booting stages. Heat degree days (HDDs) were utilized to quantify the effects of HS on NSC accumulation and translocation, revealing that the flowering stage was the most affected. These findings suggest that severe HS makes the stem the primary carbohydrate storage sink, and alleviation under combined HS aids in evaluating NSC accumulation, benefiting breeders in developing heat-tolerant rice varieties.

## 1. Introduction

Rice (*Oryza sativa* L.) is an important staple crop globally, and its production is threatened by the increasing frequency and severity of high-temperature stress due to climate change [1,2]. Studies on climate change have shown that even short episodes of heat stress (HS) during the reproductive growth stage can significantly reduce cereal crop yields, particularly for rice [3,4]. When temperatures exceed a certain threshold and persist for a specified period of time, crop growth processes are impaired, and irreversible damage to crop plants occurs due to HS [5]. The susceptibility and intensity of stress depend on the plant’s growth stage and physiological processes [6]. Point stresses or short-term heat stresses during the reproductive growth phases, particularly during booting and flowering, have the greatest impact on grain yield, and the threshold temperatures for initiating booting and flowering are identified as 33 °C and 35 °C, respectively, as noted in previous studies [6,7,8,9,10,11]. Therefore, how plants respond to high-temperature events at different growth stages can significantly affect the development of crop stress resistance.

When ambient CO_2_ is assimilated, it generates photosynthates (starch and sugars), which are then transported to the rest of the plant via the phloem [12]. For a plant to grow, it must proficiently allocate carbohydrates and ensure the effective transport and distribution of carbon from source organs to sink organs, all while enduring both biotic and abiotic challenges [13,14]. Soluble sugars and starch primarily serve as the main storage forms of nonstructural carbohydrates (NSCs) in the stem, predominantly prior to heading. Pre-heading carbohydrate reserves are acknowledged as a crucial source of carbohydrates for the grain-filling process, as they are transported from the stems to the grains, effectively substituting the newly assimilated carbohydrates [15,16]. Approximately 10–20 days post-heading, NSCs exhibit a swift transfer from stems to grains, as observed by [17], ultimately accounting for a significant portion, typically ranging from 10 to 40%, of the final grain weight, as documented by [15]. However, various factors, such as environmental conditions and genetics, can influence carbohydrate storage and transportation [18]. Stem NSC accumulation and remobilization are crucial factors affecting grain yield in unfavorable climatic conditions such as low radiation and water scarcity, which is why rice breeders are interested in them regarding yield stability in fluctuating environments [19,20].

Recent researchers have suggested that heat-tolerant rice cultivars can be developed through rice breeding by increasing the stem NSC concentration near the heading under high temperatures, which can reduce the yield loss caused by HS [21,22]. While previous studies focused on the effects of high temperatures at specific stages of rice growth, high temperatures can occur multiple times throughout a single rice growing season, and short-term HS at different growth stages can affect the accumulation of NSCs in rice [6]. Zhen et al. [7] found that HS during booting had no effect on the photosynthetic rate but reduced the sink size, inhibiting NSC translocation from the stem to the panicles and increasing carbohydrate accumulation in vegetative organs. Previous studies on wheat have shown that heat priming during earlier growth stages can significantly reduce the loss of grain production caused by HS during later reproductive stages by increasing photosynthesis and enhancing glucose remobilization from stems to grains [8,23,24,25]. Hence, it is imperative to undertake a comprehensive investigation into the impacts of recurrent HS episodes throughout the growth phase on the process of NSC remobilization.

In this research, two distinct rice cultivars, each varying in their heat tolerance, were subjected to brief periods of short-term HS at the booting and flowering stages, as well as in combined stages (HS at booting and HS at flowering). The primary aim of this study was to achieve three objectives: (1) to investigate the influence of HS intensity and duration on the dynamics of NSCs in rice leaves, stems, and panicles during the booting, flowering, and combined stages; (2) to investigate whether subjecting rice to HS during the combined (booting + flowering) stages could enhance the mobilization of stem reserves to grains, consequently leading to improved starch accumulation in the grains; and (3) to assess how short-term HS at various growth stages (booting, flowering, and combined) affects the accumulation and translocation of NSCs and their relationship with grain yield.

## 2. Results

### 2.1. Dynamics of NSCs Concentration in Leaves

**Figure 1:**
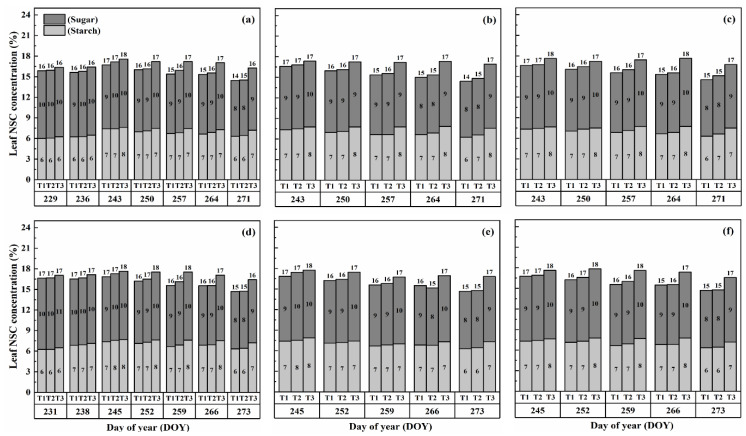
Effects of short-term heat stress with Tmax/Tmin values of T1 corresponding to 22 °C/32 °C, T2 to 30 °C/40 °C, and T3 to 34 °C/44 °C on the dynamics of NSC concentration in the leaves of HD-5 (**a**–**c**) and WYJ-24 (**d**–**f**) at booting, flowering and combined stages (booting + flowering) across 2 days (D1) in growing season 2016, respectively.

**Figure 2:**
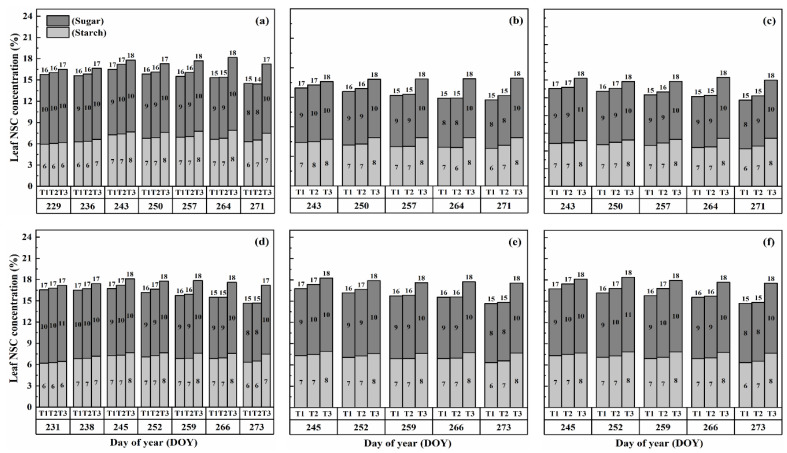
Effects of short-term heat stress with Tmax/Tmin values of T1 corresponding to 22 °C/32 °C, T2 to 30 °C/40 °C, and T3 to 34 °C/44 °C on the dynamics of NSC concentration in the leaves of HD-5 (**a**–**c**) and WYJ-24 (**d**–**f**) at booting, flowering, and combined stages (booting + flowering) across 4 days (D2) in growing season 2016, respectively.

The nonstructural carbohydrate (NSC) concentration in leaves remained relatively stable during the developmental stages, unlike stems and panicles, as shown in Figure 1 and Figure 2. However, both cultivars exhibited a gradual increase in NSC concentration in leaves with increasing temperature and duration. Notably, higher NSC concentrations were observed during the flowering stage under high-temperature treatments than in the combined and booting stages, and the highest leaf NSC concentration was consistently observed in T3D2 during grain filling.

The application of heat treatments resulted in a notable rise in starch concentration within the leaves. Specifically, at maturity, heat treatments T2 and T3 during the booting, flowering, and combined stages resulted in a substantial increase in starch concentration in leaves by 2.3–18.6%, 5.3–28.6%, and 2.3–18.6%, 5.3–28.6%, and 5.2–28.0% for Huaidao-5 (HD-5) and 1.6–18.1%, 2.4–21.4%, and 1.5–20.9% for Wuyunjing-24 (WYJ-24), respectively, compared to the control (T1). The flowering stage was the most affected, followed by the combined and booting stages, and T3 treatments showed a greater increase in starch concentration than T2 treatments. The negative impact of heat stress (HS) on starch concentration was more pronounced in HD-5 than in WYJ-24.

Furthermore, the sugar concentration in leaves also increased gradually with rising temperature and duration, except for the T2 treatment level at maturity, which showed a decrease in sugar concentration, except for HS at flowering and combined stages for HD-5. After HS treatment, sugar concentration increased more in the combined stages than in the flowering and booting stages. At maturity, under the T3D1 and T3D2 treatments during the booting, flowering, and combined stages, there was a notable rise in sugar concentration of 11.2–19.2%, 14.8–22.6%, and 12.6–19.4% for HD-5, and 9.9–16.6%, 13.5–18.4%, and 12.6–18.1% for WYJ-24, respectively, compared to the control (T1). The flowering stage exhibited the highest sensitivity to heat stress, followed by the combined and booting stages, respectively.

### 2.2. Dynamics of NSC Concentration in Stems

**Figure 3:**
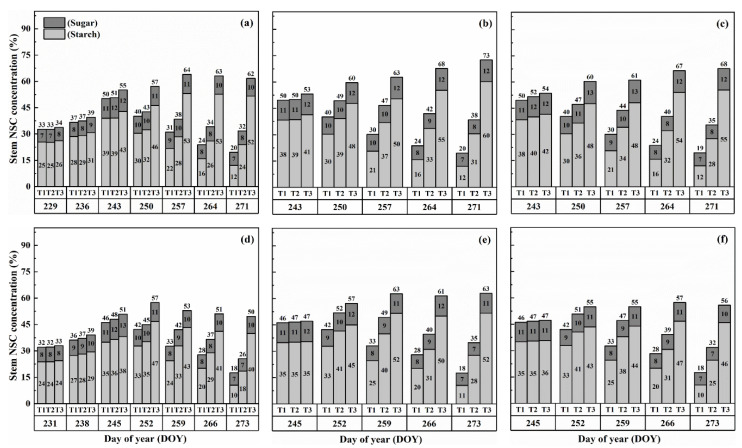
Effects of short-term heat stress with Tmax/Tmin values of T1 corresponding to 22 °C/32 °C, T2 to 30 °C/40 °C, and T3 to 34 °C/44 °C on the dynamics of NSC concentration in the stems of HD-5 (**a**–**c**) and WYJ-24 (**d**–**f**) at booting, flowering, and combined stages (booting + flowering) across 2 days (D1) in growing season 2016, respectively.

**Figure 4:**
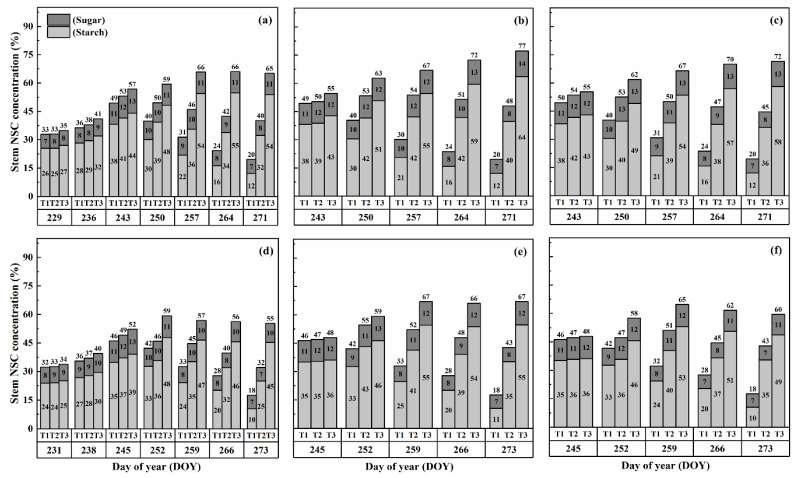
Effects of short-term heat stress with Tmax/Tmin values of T1 corresponding to 22 °C/32 °C, T2 to 30 °C/40 °C, and T3 to 34 °C/44 °C on the dynamics of NSC concentration in the stems of HD-5 (**a**–**c**) and WYJ-24 (**d**–**f**) at booting, flowering, and combined stages (booting + flowering) across 4 days (D2) in growing season 2016, respectively.

The concentration of nonstructural carbohydrates (NSCs) in the stems exhibited distinct patterns throughout the growth stages. Initially, under normal conditions (T1), NSC concentration in the stems increased and reached its peak near the flowering stage. Subsequently, during the grain-filling process, NSC concentration declined. However, under HS conditions, the stem NSC concentration continued to rise significantly as the high-temperature level and duration of HS increased for both cultivars (Figure 3 and Figure 4). At maturity, WYJ-24 exhibited lower stem NSC concentration compared to HD-5.

Furthermore, the starch concentration in the stems also showed a significant increase following heat treatment. At maturity, the starch concentration in the stems during booting, flowering, and combined stages of HS increased by 98.2–364.4%, 152.9–426.6%, and 129.0–380.2% for HD-5, and 76.4–332.7%, 162.7–420.4%, and 135.2–374.6% for WYJ-24 under T2 and T3, respectively, compared to the control (T1). These findings indicate that the HS had the most pronounced impact during the flowering stage, with the combined and booting stages following as the subsequent most affected stages. Additionally, the increase in starch concentration was more pronounced under T3 than in T2. Notably, HS had a more detrimental impact on starch concentration in the stems of HD-5 compared to WYJ-24.

Conversely, the sugar concentration in the stems gradually increased with rising temperature and duration. Immediately after HS treatment, the sugar concentration showed a more substantial increase during the combined stages compared to the flowering and booting stages. For high temperatures during booting, flowering, and combined stages, the stem sugar concentration under T2 and T3 increased by 3.6–50.6%, 6.2–82.0%, and 5.1–79.1% for HD-5, and 0.3–42.2%, 1–73.8%, and 0.6–54.1% for WYJ-24, respectively, compared to the control (T1). The increase in sugar concentration was most sensitive to HS during the flowering stage, followed by the combined and booting stages, respectively.

### 2.3. Dynamics of NSC Concentration in Panicles

**Figure 5:**
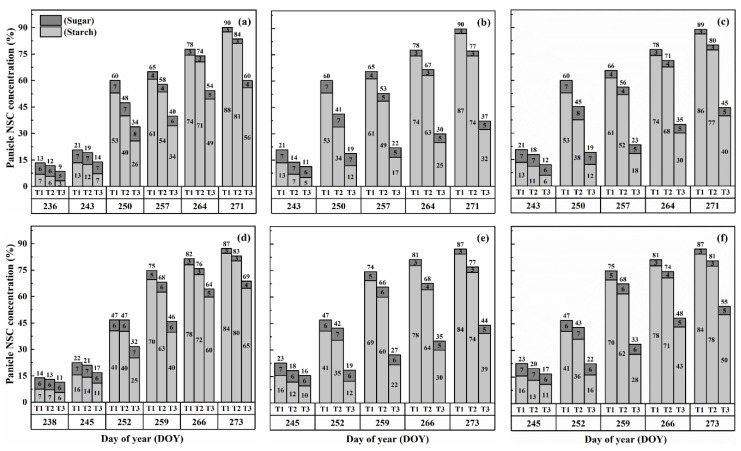
Effects of short-term heat stress with Tmax/Tmin values of T1 corresponding to 22 °C/32 °C, T2 to 30 °C/40 °C, and T3 to 34 °C/44 °C on the dynamics of NSC concentration in the panicles of HD-5 (**a**–**c**) and WYJ-24 (**d**–**f**) at booting, flowering, and combined stages (booting + flowering) across 2 days (D1) in growing season 2016, respectively.

**Figure 6:**
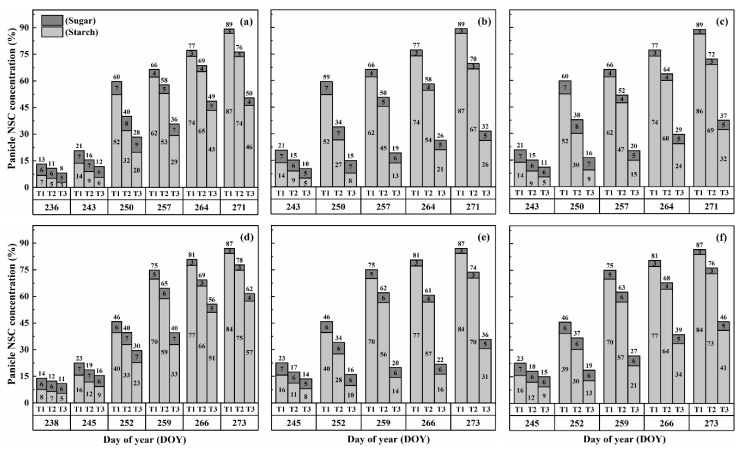
Effects of short-term heat stress with Tmax/Tmin values of T1 corresponding to 22 °C/32 °C, T2 to 30 °C/40 °C, and T3 to 34 °C/44 °C on the dynamics of NSC concentration in the panicles of HD-5 (**a**–**c**) and WYJ-24 (**d**–**f**) at booting, flowering and combined stages (booting + flowering) across 4 days (D2) in growing season 2016, respectively.

The concentration of nonstructural carbohydrates (NSCs) in the panicles exhibited distinct patterns during the grain-filling process. Overall, panicle NSC concentration increased gradually, indicating the accumulation of carbohydrates in the panicles (Figure 5 and Figure 6). However, when subjected to HS at the booting, flowering, and combined stages, the panicle NSC concentration in both cultivars significantly decreased with increasing temperature levels and durations. Significantly, the lowest NSC concentration in the panicle was consistently observed during the grain-filling process when subjected to the T3D2 treatment, with the flowering stage being the most adversely affected.

Similarly, the starch concentration also decreased significantly after heat treatments for all stages. At maturity, it was observed that under T2 and T3 treatments during booting, flowering, and combined stages, there were significant reductions in the starch concentration, ranging from 7.5–46.8%, 14.7–69.8% and 10.6–62.5% for HD-5 and 5.0–31.9%, 12.3–63.7% and 8.1–51.0% for WYJ-24, in comparison to the control (T1). These results revealed that flowering was the most affected stage, followed by the combined and booting stages, and the decrease in starch concentration was much greater under T3 than under T2. It was observed that HS had more negative effects on the starch concentration of HD-5 than WYJ-24.

However, the sugar concentration in the panicles first decreased after heat treatment but gradually increased with the rising temperature and duration, but this increase was not as significant as the decrease in starch concentration. The results at maturity showed that under T2 and T3 treatments at booting, flowering and combined stages led to the 1.1–66.9%, 10.3–105.2% and 8.5–96.1% increase in the sugar concentration in HD-5 and −1.3–51.3%, 8.9–81.2% and 5.9–72.2% increase in the sugar concentration in WYJ-24, respectively, as compared to the control (T1). The increase in sugar concentration was more sensitive to flowering HS than combined and booting stages, respectively.

**Figure 7:**
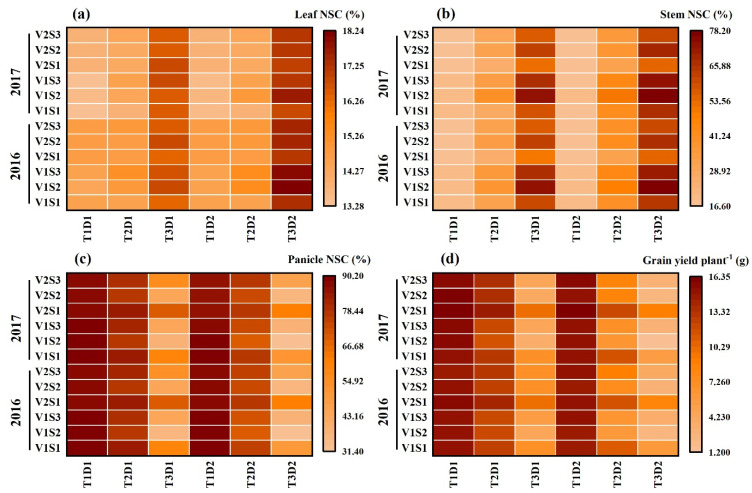
Effects of short-term heat stress with Tmax/Tmin values of T1 corresponding to 22 °C/32 °C, T2 to 30 °C/40 °C, and T3 to 34 °C/44 °C on the NSC concentrations during physiological maturity in leaf (**a**), stem (**b**), panicle (**c**), and grain yield (**d**) in HD-5 [V1] and WYJ-24 [V2] at booting [S1], flowering [S2], and the combined stages (booting + flowering) [S3] during 2-day (D1) and 4-day (D2) stress periods.

The NSC concentration (%) in leaves and stems reached its peak at maturity under treatment T3D2, whereas the NSC concentration (%) in panicles at maturity was notably affected by T3D2, with flowering being the stage showing the most significant impact. Notably, Cultivar HD-5 displayed higher susceptibility compared to WYJ-24 under high-stress conditions (Figure 7).

### 2.4. Effect of Heat Stress on NSC Accumulation at Maturity

The accumulation of NSCs in the leaves and stems at maturity increased with higher temperature levels and longer duration of heat stress, except in treatment T2D1, where NSC accumulation in the leaves of WYJ-24 decreased (Table 1 and Table 2). Generally, HD-5 exhibited higher NSC accumulation in leaves and stems compared to WYJ-24. However, both varieties showed decreased NSC accumulation in panicles.

During grain filling, HS at the booting, flowering, and combined stages significantly increased NSC accumulation in stems. Remarkably, under severe HS treatments (T3), the NSC accumulation in stems continued to increase instead of decreasing. Moreover, at maturity, compared to the control (T1), NSC accumulation in stems at the booting, flowering, and combined stages under T2 and T3 temperature levels increased by 103.3–548.6%, 153.6–708.3%, and 120.1–684.9% for HD-5, and 72.0–432.1%, 143.8–701.9%, and 123.3–678.6% for WYJ-24, respectively. These results indicate that severe HS prevents NSC translocation from stems to developing panicles, leading to the re-accumulation of newly assimilated NSCs in stems.

As shown in Table 1 and Table 2, the NSC accumulation in panicles decreased with higher temperature levels and longer durations of HS for both cultivars. Additionally, lower NSC accumulation in panicles was observed under high-temperature treatments during flowering compared to combined and booting stages, respectively. At maturity, compared to the control (T1), the NSC accumulation in panicles at the booting, flowering, and combined stages under T2 and T3 temperature levels declined by 20.3–78.5%, 32.1–95.4%, and 28.2–90.9% for HD-5, and 10.6–58.4%, 24.2–92.8%, and 17.7–88.2% for WYJ-24, respectively. It was observed that the high-temperature level had a more negative impact on NSC accumulation in panicles of HD-5 compared to WYJ-24.

Furthermore, at maturity, the NSC accumulation in leaves at the booting, flowering, and combined stages under T2 and T3 temperature levels increased by 3.7–39.3%, 1.5–41.2%, and 4.0–44.4% for HD-5, and −0.3–39.1%, −1.7–39.5%, and −0.7–43.1% for WYJ-24, respectively, compared to the control (T1). These results indicate that under heat stress, the increase in NSCs in leaves was more significant in HD-5 compared to WYJ-24.

### 2.5. Effect of Heat Stress on NSC Translocation at Maturity

As the temperature and duration rose, there was a simultaneous decrease in both the translocation of NSCs and the efficiency of NSC translocation in stems. At maturity, averaged across both years, NSC translocation at booting, flowering, and combined stages under T2 and T3 temperature levels decreased by 68.9–377.1%, 102.0–487.9% and 79.9–470.8% for HD-5 and 39.6–238.4%,79.5–396.9% and 68.1–383.5% for WYJ-24, respectively, as compared to the control (T1). In addition, NSC translocation efficiency at booting, flowering, and combined stages has the same trend as the decrease in translocation as compared to the control (T1).

It was observed that as temperature levels and durations increased, both NSC translocation and its efficiency in stems decreased. At maturity, averaged across both years, the translocation of nonstructural carbohydrates (NSCs) during the booting, flowering, and combined stages under T2 and T3 temperature levels exhibited a substantial decrease. For HD-5, the decreases ranged from 68.9% to 377.1%, 102.0% to 487.9%, and 79.9% to 470.8%, respectively, compared to the control (T1). Similarly, for WYJ-24, the decreases ranged from 39.6% to 238.4%, 79.5% to 396.9%, and 68.1% to 383.5%, respectively, as compared to the control (T1). Likewise, the efficiency of NSC translocation at the booting, flowering, and combined stages followed the same decreasing trend compared to the control. Notably, under extreme high-temperature stress (T3D1 and T3D2), NSC translocation and efficiency turned negative, implying that NSCs were not translocated to the panicles rather than re-accumulated in the stem (Table 1 and Table 2).

Moreover, the research investigated the impact of the contribution of NSCs in stem tissues to grain yield prior to heading. The results indicated a decrease in NSC contribution as temperature and the duration of heat stress increased. On average, for HD-5, NSC contribution to grain yield at the booting, flowering, and combined stages ranged from 19.5% to −143.1%, −684.9%, and −336.9%, and for WYJ-24, the range was from 18.4% to −46.7%, −341.9%, and −302.9%, respectively. In most treatments, WYJ-24 exhibited greater NSC translocation, NSC translocation efficiency, and a more substantial contribution of stem NSC to grain yield compared to HD-5, suggesting that WYJ-24 had enhanced capacity for NSC translocation amid heat stress. Furthermore, it was observed that flowering was the growth stage most severely affected by heat stress, followed by the combined and booting stages.

### 2.6. Quantification of the Effects of Heat Stress on NSC Accumulation

**Figure 8:**
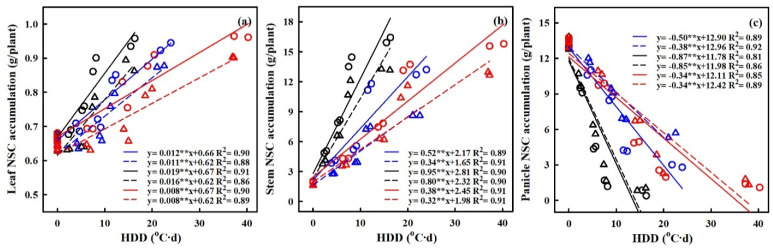
Relationships of NSCs accumulation of leaf (**a**), stem, (**b**) and panicle (**c**) with heat degree days (HDDs) in 2016–2017. ○ and solid line indicates HD-5, ∆ and dashed line indicates WYJ-24; blue, black, and red lines are for booting, flowering, and combined stages, respectively. ⁎⁎ indicates significant difference at *p* < 0.01.

The quantification of the effects of HS on the accumulation of nonstructural carbohydrates (NSCs) was carried out, with a focus on the relationship between heat degree days (HDDs) and NSC accumulation (refer to Figure 8). HDDs, which integrate both the intensity and duration of elevated temperatures, demonstrated stronger correlations with grain yield and NSC accumulation compared to other metrics. The results showed a significantly negative relation between HDDs and NSC accumulation in panicles, while the relations between HDDs and NSC accumulation in leaves and stems were significantly positive.

With every 1 °C-day increase in HDDs, NSC accumulation in panicles decreased by 49.6%, 38.2%, and 86.8%, and 85.2%, 34.3% and 33.5% for HD-5 and WYJ-24 at the booting, flowering, and combined stages, respectively. This suggests that the largest loss of NSC accumulation in panicles occurred during the flowering stage, followed by the combined and booting stages and that HD-5 was more adversely affected than WYJ-24. Additionally, NSC accumulation in stems showed an increasing trend with rising temperatures. For every 1 °C-day increase in HDDs, NSC accumulation in the stems increased by 51.7%, and 34.3%, 95.2%, and 80.2% and 38.0% and 32.4% for HD-5 and WYJ-24 at the booting, flowering, and combined stages, respectively. Furthermore, for NSC accumulation in leaves, there was an increase of 1.2%, 1.9%, and 0.8% for HD-5, and 1.1%, 1.6% and 0.8% for WYJ-24, with every 1 °C-day increase in HDD at the booting, flowering, and combined stages.

**Figure 9:**
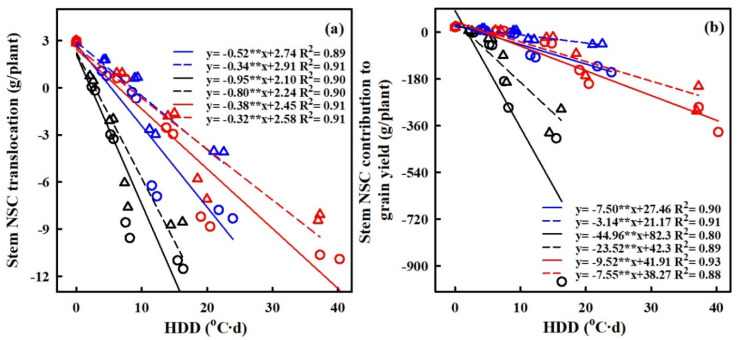
Relationships of stem NSC translocation (g/plant) (**a**), stem NSC contribution to grain yield (g/plant) (**b**) and with heat degree days (HDDs) in 2016–2017. ○ and solid line indicates HD-5, ∆ and dashed line indicates WYJ-24; blue, black, and red lines are for booting, flowering, and combined stages, respectively. ⁎⁎ indicates significant difference at *p* < 0.01.

**Figure 10:**
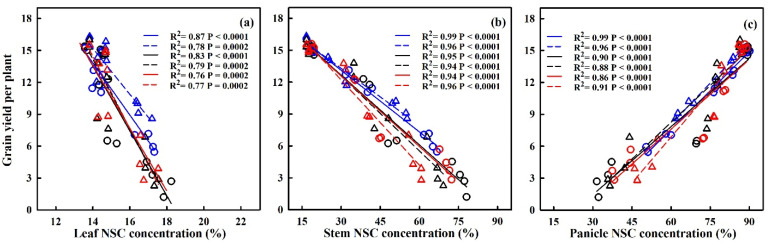
Relationships between NSC concentration in leaves (**a**), stems (**b**) and panicles (**c**) at maturity with grain yield in 2016–2017. ○ and solid line indicates HD-5, ∆ and dashed line indicates WYJ-24; blue, black, and red lines are for booting, flowering, and combined stages, respectively.

The translocation of NSCs in stems and the contribution of stem NSCs to grain yield showed significant negative correlations with HDD (refer to Figure 9). For every 1 °C-day increase in HDD, stem NSC translocation decreased by 51.7%, 34.3%, and 95.2%, and 80.2%, 38.1% and 32.4% for HD-5 and WYJ-24 at the booting, flowering, and combined stages, respectively. Despite the higher HDDs at the combined stages, the effect of heat stress was less pronounced compared to the flowering and booting stages, indicating some alleviation during the combined stages. Similarly, the contribution of stem NSCs to grain yield also exhibited a negative correlation with HDDs. Furthermore, the relationship between grain yield and NSC concentration in leaves, stems, and panicles was analyzed (refer to Figure 10). The analysis uncovered a notable positive association between grain yield and NSC concentration in leaves and stems, alongside a significant negative correlation between grain yield and panicle NSC concentration.

## 3. Discussion

### 3.1. Effect of Heat Stress on NonStructural Carbohydrate Dynamics and Remobilization in Plant Parts

Efficient assimilate remobilization from vegetative tissues to grains is pivotal for rice yield development, especially under adverse environmental conditions [26,27]. The impact of elevated temperatures on rice nonstructural carbohydrate (NSC) remobilization hinges not only on temperature levels and growth stages but also on the frequency of high-temperature events [7,28].

Upon exposure to heat stress, notable shifts in sugar and starch concentrations were observed across different plant parts. Leaves and stems displayed increased sugar and starch concentrations, while panicles exhibited decreased starch concentration and increased sugar concentration, consistent with previous findings [7,22]. The decrease in starch accumulation in panicles after heat stress (HS) during booting and flowering stages might be attributed to reduced activity of the soluble starch synthase enzymes like invertase, SUS, and AGPase, which play crucial roles in sucrose-to-starch conversion and translocation, thereby affecting its subsequent deposition in grains [29,30,31]. While under normal conditions, at the maturity stage in rice, the concentration of NSCs in the stems ranged from 10.1% to 31.6% [13,26], which is consistent with the NSC concentration of our control treatments. Intriguingly, under severe heat stress, the NSC concentration and accumulation in stems and leaves increased, suggesting impaired translocation to developing panicles and subsequent re-accumulation in stems. Thus, stems effectively acted as new carbohydrate storage sites during grain filling. Additionally, HS led to a decrease in the final NSC concentration in panicles (Figure 5 and Figure 6), and the panicle NSC concentration remained consistently low throughout the grain-filling process under severe heat stress. This indicates that the carbohydrates were unable to be translocated from the stems to the panicles, aligning with previous studies investigating the effects of HS [7,27,28,32]. Based on our study, poor NSC translocation does not appear to be attributed to assimilates availability. This emphasizes how HS during booting and flowering stages directly impacts carbon assimilate utilization and distribution, particularly in heat-sensitive rice cultivars [33,34].

Several factors influence stem nonstructural carbohydrate (NSC) translocation, including source–sink relationships, sink activity, vascular bundle characteristics, and phloem loading and unloading processes [26,35,36]. HS during booting and flowering stages reduces sink size, affecting carbohydrate translocation and grain development [7,22]. Sugars, rather than phytohormones, appear to regulate the source–sink relationship under HS in rice [22]. Vascular bundle blockage and inadequate phloem unloading caused by HS can also decrease translocation [22,36]. Comparisons of different stages revealed that the translocation of NSCs was most significantly affected by HS during the flowering stage, followed by the combined and booting stages. These findings suggest that the pre-heat treatment during the booting stage conferred resilience to plants, enabling them to withstand HS during the flowering stage. This finding aligns with the study by [23,37], which demonstrates that pre-anthesis heat acclimation improves the mobilization of stored stem reserves into growing grains by enhancing the activity of fructan-catalyzing enzymes, such as SST and FFT.

The lack of NSC contribution to grain yield prior to heading in the T2D2, T3D1, and T3D2 treatments suggests that NSCs from the stems were not effectively translocated to the grains. These findings further highlight the reliance on newly assimilated carbohydrates for grain yield during extreme HS [7]. Interestingly, our results reveal a higher accumulation of NSCs in the stems and a lower conversion of NSCs to panicles during the flowering, combined, and booting stages. Additionally, flowering was found to be the stage most affected by heat stress, followed by the combined and booting stages. This suggests that implementing a pre-heat treatment during the booting stage has the potential to enhance the mobilization of NSCs from the stems to the panicles when experiencing HS during the flowering stage. It is evident that heat-acclimated plants exhibit enhanced photosynthetic capacity, characterized by increased green flag leaf area, elevated chlorophyll and soluble protein contents, improved photosynthesis, and more efficient antioxidant enzyme activities [38,39]. These improvements in source productivity result in a more abundant carbohydrate supply and reduced grain yield loss compared to plants subjected to HS at a single stage. Moreover, Zhang et al. [40] explained that the pretreated plants with HS exhibited activated stress signaling processes and up-regulated stress-related genes, including heat-shock proteins and osmotins, resulting in enhanced thermo-tolerance. This was achieved through the modification of gene expressions, which in turn up-regulated physiological processes like photosynthesis and antioxidation, ultimately enhancing grain yield.

Previous studies have extensively investigated the heat tolerance of rice cultivars, primarily focusing on the seed-setting rate [41,42]. However, recent research has taken a novel approach by examining the heat tolerance of rice cultivars in terms of carbohydrate utilization [22,27]. Shi et al. [43] have proposed that acclimation, as a strategy to combat heat stress, may be more successful in cultivars with a higher tolerance, such as WYJ-24, which is consistent with our study. Our findings reveal that the heat-tolerant cultivar, WYJ-24, exhibits enhanced remobilization and accumulation of nonstructural carbohydrates (NSCs) in the panicles compared to HD-5. This observation is in line with the results reported by Zhen et al. [7] and supports the notion that WYJ-24 possesses superior NSC translocation characteristics. Collectively, these findings suggest that the translocation of NSCs operates with greater efficiency in heat-resistant cultivars than in heat-sensitive ones when subjected to high-temperature conditions.

### 3.2. Quantifying the Impact of Heat Stress

Over the last few years, there has been an increase in the use of quantitative methods in research studies to explore the impact of HS on both the physiological performance and crop yield of rice [11,42,44,45]. However, there is still a need to improve the accuracy of these quantitative algorithms for predicting the effects of HS. Additionally, limited research has specifically examined the physiological responses and yield of rice under HS at multiple critical stages. The heat degree days (HDDs) index, which combines temperature duration and intensity, has been utilized to comprehensively evaluate the impact of HS [11,42,46]. Post-anthesis, NSC accumulation showed a linear increase in leaves and stems but a linear decrease in panicles with increasing HDD in both rice cultivars. Flowering emerged as the most heat-susceptible stage, leading to sterility and a loss of sink capacity, which could explain the reduced NSC accumulation in panicles and increased accumulation in stems during HS at the flowering stage compared to other stages [45]. Moreover, HD-5 and WYJ-24, the two rice cultivars, demonstrated varying susceptibility to heat stress, with HD-5 showing higher vulnerability to NSC loss in panicles compared to WYJ-24. These findings highlight the importance of understanding cultivar-specific responses to HS for breeding heat-tolerant varieties. Consistent with previous research by [7,22], we also found a negative relationship between HDD and panicle NSC accumulation, while stem NSC accumulation exhibited a positive relationship during HS at the booting and flowering stages. These findings suggest that a greater level of NSCs in the stems at maturity, resulting from poor translocation into grains, hampers yield formation. These findings highlight the intricate interaction between HS and carbohydrate metabolism in rice, providing valuable insights for climate-resilient crop management strategies to ensure food security amidst increasing global temperatures.

Additionally, our study investigated the post-anthesis translocation of NSCs from stems to panicles, which exhibited a linear decrease with increasing HDD (Figure 8). This reduction in stem NSC translocation could limit the availability of energy reserves required for grain filling, resulting in decreased grain yield under HS at booting, flowering, and combined stages in both cultivars. However, we observed some alleviation of HS impact during the combined stages despite higher HDDs, suggesting potential adaptive responses or compensatory mechanisms in rice plants [23,25,43]. Thus, when considering critical developmental stages, the use of HDD is crucial for predicting NSC concentration, translocation, and yield under heat stress. The insights gained from the relationship between grain yield and NSC concentrations at maturity, as well as the association between NSC accumulation, stem NSC translocation, and HDDs in our study, can be seamlessly incorporated into established crop simulation models. This integration holds the potential to significantly advance the accuracy of grain yield and quality predictions during the reproductive stages under high-temperature stress under forthcoming climate conditions.

## 4. Materials and Methods

### 4.1. Experimental Details

The experiment was carried out in Jiangsu Province, China, specifically in Rugao City, located at 120.33° E and 32.23° N. The study employed two cultivars of japonica rice, namely Wuyunjing-24 (WYJ-24) and Huaidao-5 (HD-5), and was carried out between 2016 and 2017, utilizing an environmentally regulated plant phytotron. Plastic pots were utilized for the transplantation of the rice seedlings, with 66 plants per square meter, a density like that of japonica rice in the local vicinity. The pots were 35.6 cm in diameter and 29.8 cm in height and contained 22.4 L of soil. The soil composition in this area boasts a clay loam texture enriched with organic matter, measuring 15.2 grams per kilogram. Within the 0–30 cm soil layer, essential nutrients are present, with 1.1 grams per kilogram of available nitrogen (N), 13.4 milligrams per kilogram of available phosphorus (P), and 72.9 milligrams per kilogram of available potassium (K). Additionally, the soil exhibits a pH value of 8.16, indicating its alkaline nature. Base fertilizers of 1.5 g N, 1.5 g P_2_O_5_, and 2 g K_2_O were applied to each pot before transplanting, with supplemental N added during the mid-tiller and jointing stages. Local rice production standards were followed for irrigation and crop protection practices.

**Figure 11:**
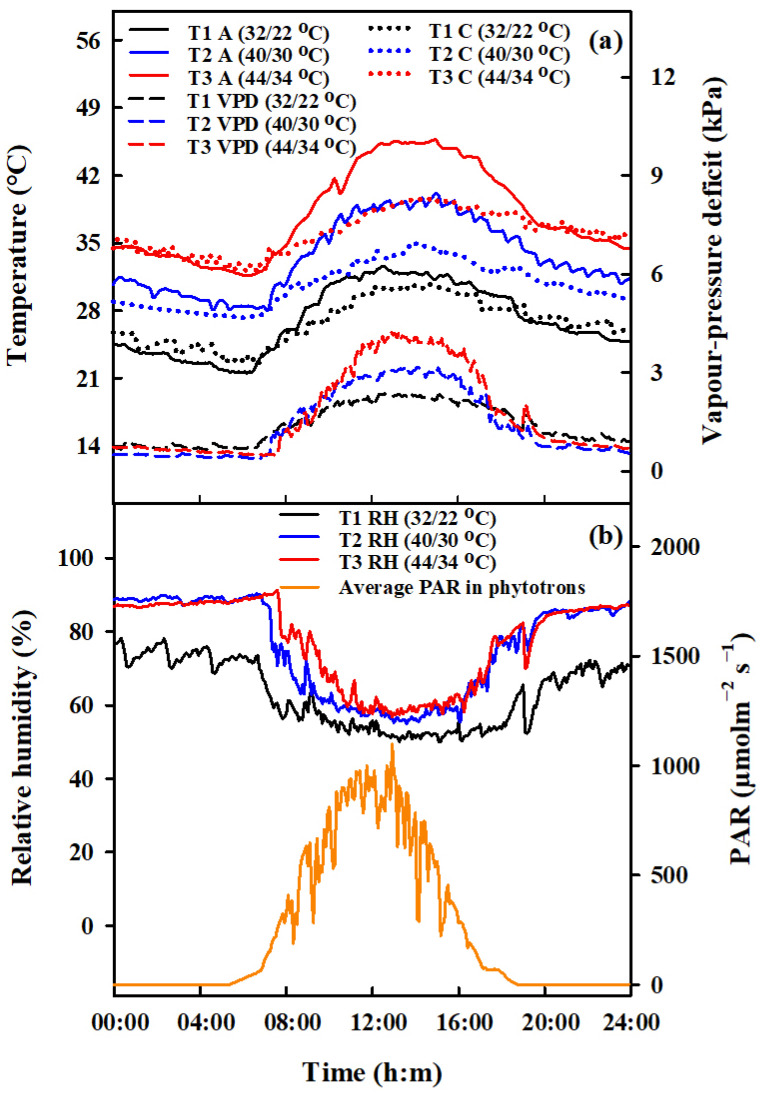
Design of the daily dynamics of temperature, vapor pressure deficit, relative air humidity and photosynthetically active radiation in the environment-controlled phytotron. Diurnal variations of air (A, °C, solid lines, (**a**) left vertical axis), and canopy temperature (C, °C, dotted lines, (**a**) left vertical axis), vapor pressure deficit (VPD, kPa, dashed lines, (**a**) right vertical axis), relative air humidity (RH, %, solid lines, (**b**) left vertical axis), and average photosynthetically active radiation (PAR, μmol m^−2^s^−1^, (**b**) right vertical axis) in phytotrons during heat stress treatments in 2016.

Apart from the periods when heat stress (HS) was applied, all pots were kept under standard ambient conditions. Pots containing an equal number of tillers were moved to phytotron chambers (which were 3.4 m long, 3.2 m wide, and 2.8 m high) for HS treatments during the booting, flowering, and combined stages. During the combined stage, rice plants that had already been heat-stressed during the booting stage were moved back to the phytotron for further HS treatment during the flowering stage. HS treatments involved three temperature levels (T1, T2, T3), with daily maximum and minimum ranges of 22 °C/32 °C, 30 °C/40 °C and 34 °C/44 °C, respectively (Table 3). The duration of these treatments was two and four days, but for the combined stage treatment, the duration was extended to four and eight days. The temperature in the phytotrons was simulated to mimic the dynamics of ambient temperature, and these were consistent across the two years studied. The data presented here reflects the trend observed in 2016 (Figure 11).

The vapor pressure deficit (VPD) values were determined using the calculation method proposed by [47]. In the phytotrons, various sensors from METER Group, Inc. ( Washington, DC, USA) (including PYR solar radiation sensors, 5TM sensors, and VP-3 sensors) were installed to measure the photosynthetically active radiation (PAR, µmol m^−2^ s^−1^, air temperature (Ta, °C), soil temperature (Ts, °C), and relative humidity (RH, %), respectively. To assess the canopy temperature (Tc, °C), an infrared radiometer (SI-111, Apogee Instruments, Logan, UT, USA) was positioned at the center of each chamber, placed 1.6 m above the ground. The radiometer was angled diagonally downwards to cover a 0.8 m^2^ section of the plant surface. Figure 11 presents the variations observed in these meteorological parameters.

### 4.2. Measurement of Grain Yield Parameters

Upon reaching maturity, a random selection of five rice pots was made from each treatment, which was replicated three times, resulting in a total of 15 pots for the assessment of grain yield per plant (YPP, in grams per plant). The T2 and T3 treatments resulted in extreme spikelet sterility, resulting in the translocation of carbon and nitrogen nutrients toward the axillary buds and roots. Consequently, new panicle-bearing tillers, termed regenerated tillers, emerged from the axillary buds. The ultimate panicles in the T2 and T3 treatments consisted of a combination of original and regenerated tillers. This suggests that both types of tillers (original and total, including original + regenerated tillers) contributed to grain yield and its components. The calculations for yield and yield components encompassed the contribution of regenerated tillers.

### 4.3. Calculation of NSC Accumulation and Translocation Parameters

Rice plants were sampled at intervals of 6–7 days, covering the period from the heading stage to physiological maturity. For each designated time point, three pots per treatment were selected meticulously, resulting in three independent replicates. These rice samples were then separated into leaves, stems (including leaf sheaths and culms), and panicles. Subsequently, the dry weights of these plant components were determined by subjecting them to a forced-air oven set at 85 °C for 72 h. The collected samples underwent careful grinding using a steel grinder to assess the concentration of nonstructural carbohydrates (NSCs). The quantification of NSC concentration, which includes sugars and starch, in different plant parts (leaf, stem, and panicle) followed the methodologies described by [48], employing the anthrone-sulfuric acid method. Initially, total soluble sugars were extracted using 80% ethanol, followed by starch extraction through perchloric acid digestion.

For the analysis of NSC (nonstructural carbohydrate) distribution across different plant components, the NSC concentration was calculated by multiplying it with the corresponding dry matter content. Subsequently, the translocation of NSCs within the plant was evaluated by comparing the variations in NSC accumulation between the heading and physiological maturity stages, with particular emphasis on the stems. The efficiency of NSC translocation was quantified by determining the proportion of NSCs translocated from the stems to the total NSCs accumulated during the heading stage. Furthermore, the impact of NSCs stored in the stems on grain yield was investigated by analyzing the ratio of NSC translocation to grain yield.

### 4.4. Statistical Analysis

To assess treatment differences, Duncan’s new multiple-range tests were applied at a significance level of 0.05. Furthermore, a simple linear regression analysis was conducted to investigate the correlation between heating degree days (HDDs) and the accumulation of NSCs in leaves, stems, and panicles. This analysis aimed to comprehensively evaluate the influence of varying high-temperature levels and durations on NSC accumulation. Additionally, a basic linear regression model was employed to determine the relationship between grain yield and NSC concentration in leaves, stems, and panicles. The computation of HDDs followed the methodologies outlined in previous studies [8,11].

## 5. Conclusions

Compared to heat stress (HS) at a single flowering stage, subjecting plants to short-term HS during the booting stage following flowering was found to enhance the remobilization of dry matter from stems to grains. Conversely, HS during the booting, flowering, or combined stages resulted in a significant yield reduction by impeding the remobilization of NSCs. The duration and intensity of HS had a notable adverse effect on NSC remobilization before heading and its contribution to grain yield. Additionally, HS increased carbohydrate accumulation in vegetative organs and hindered NSC translocation from stems to panicles during the booting, flowering, and combined stages, with extreme HS completely halting NSC translocation. The impact of extreme HS on NSC translocation was severe, transforming the stem from a source for grain filling to a sink for carbohydrate accumulation. Heat degree days (HDDs), which account for HS severity and duration, were used to evaluate the effects on NSC accumulation. The established correlations between NSC accumulation, grain yield, and HDDs offer insights for predicting grain yields under future climate conditions. This study highlights the critical significance of accumulating high panicle NSCs in heat-tolerant rice cultivars, opening avenues for their strategic integration into rice breeding programs as a viable means to alleviate yield losses caused by heat stress.

## Figures and Tables

**Table 1:** Effects of short-term heat stress with Tmax/Tmin values of T1 corresponding to 22 °C/32 °C, T2 to 30 °C/40 °C, and T3 to 34 °C/44 °C on the NSC accumulation and translocation at booting, flowering, and combined stages (booting + flowering) in growing season 2016.

Stage	Cultivar	Treatment	NSCs Accumulation at Maturity (g/plant)			NSCs Translocation (g/plant)	NSCs Translocation Efficiency (%)	Grain Yield per Plant (g)	Contribution of Stem NSCs to Grain Yield (%)
			Leaf	Stem	Panicle				
Booting	HD-5	T1D2	0.67 b	2.02 e	13.45 a	2.90 a	58.93 a	14.88 a	19.49 a
		T2D1	0.71 b	3.85 d	10.59 b	1.07 b	21.73 b	12.69 b	8.56 ab
		T2D2	0.72 b	5.20 c	8.44 c	−0.28 c	−5.65 c	11.06 c	−2.38 b
		T3D1	0.83 ab	11.15 b	4.23 d	−6.23 d	−126.57 d	7.05 d	−88.66 c
		T3D2	0.92 a	12.70 a	3.01 e	−7.78 e	−158.16 e	5.94 e	−131.79 d
	WYJ-24	T1D2	0.63 b	1.61 d	13.06 a	2.99 a	64.99 a	15.53 a	19.33 a
		T2D1	0.64 b	2.81 cd	11.64 b	1.79 ab	38.94 ab	14.03 b	12.82 a
		T2D2	0.66 b	3.94 c	9.09 c	0.66 b	14.38 b	11.69 c	5.59 a
		T3D1	0.76 ab	7.25 b	6.91 d	−2.65 c	−57.52 c	10.03 d	−26.89 b
		T3D2	0.87 a	8.64 a	5.30 e	−4.04 d	−87.75 d	8.58 e	−47.81 c
Flowering	HD-5	T1D2	0.67 b	2.02 e	13.62 a	2.90 a	58.96 a	14.82 a	19.62 a
		T2D1	0.68 b	4.86 d	9.51 b	0.06 b	1.21 b	12.30 b	0.53 b
		T2D2	0.75 b	7.90 c	4.35 c	−2.98 c	−60.5 7c	6.25 c	−47.54 c
		T3D1	0.86 a	13.50 b	1.68 d	−8.58 d	−174.40 d	4.53 d	−189.31 d
		T3D2	0.93 a	15.91 a	0.85 d	−10.99 e	−223.33 e	2.70 e	−408.54 e
	WYJ-24	T1D2	0.63 c	1.62 e	12.90 a	2.98 a	64.83 a	14.78 a	20.19 a
		T2D1	0.64 c	4.15 d	9.64 b	0.45 b	9.81 b	12.47 b	3.54 a
		T2D2	0.64 c	6.58 c	5.61 c	−1.98 c	−43.15 c	7.62 c	−26.04 a
		T3D1	0.76 b	10.66 b	3.01 d	−6.06 d	−131.68 d	6.85 c	−89.52 b
		T3D2	0.86 a	13.15 a	1.03 e	−8.55 e	−185.97 e	2.88 d	−296.55 c
Combined	HD-5	T1D2	0.68 b	2.02 e	13.55 a	2.90 a	58.97 a	15.25 a	19.09 a
		T2D1	0.69 b	4.31 d	9.73 b	0.61 b	12.45 b	12.18 b	5.04 a
		T2D2	0.83 ab	7.46 c	4.86 c	−2.54 c	−51.65 c	6.71 c	−38.13 b
		T3D1	0.88 ab	13.13 b	2.54 d	−8.21 d	−166.87 d	5.69 c	−145.88 c
		T3D2	0.96 a	15.56 a	1.38 e	−10.64 e	−216.27 e	3.70 d	−288.91 d
	WYJ-24	T1D2	0.63 c	1.61 e	12.94 a	2.99 a	64.96 a	14.89 a	20.04 a
		T2D1	0.64 c	3.67 d	10.64 b	0.93 b	20.23 b	13.16 b	7.01 a
		T2D2	0.66 c	6.22 c	6.75 c	−1.62 c	−35.16 c	8.81 c	−18.69 b
		T3D1	0.79 b	10.39 b	3.85 d	−5.79 d	−125.81 d	7.03 d	−82.27 c
		T3D2	0.90 a	12.67 a	1.79 e	−8.07 e	−175.55 e	3.89 e	−207.83 d

In the corresponding column for each variety, distinct letters signify statistically significant differences (*p* < 0.05) among the treatments. T denotes the temperature level, and D represents the duration of heat stress. The presented means are derived from three replicates.

**Table 2:** Effects of short-term heat stress with Tmax/Tmin values of T1 corresponding to 22 °C/32 °C, T2 to 30 °C/40 °C, and T3 to 34 °C/44 °C on the NSC accumulation and translocation at booting, flowering, and combined stages (booting + flowering) in growing season 2017.

Stage	Cultivar	Treatment	NSCs Accumulation at Maturity (g/plant)			NSCs Translocation (g/plant)	NSCs Translocation Efficiency (%)	Grain Yield per Plant (g)	Contribution of Stem NSCs to Grain Yield (%)
			Leaf	Stem	Panicle				
Booting	HD-5	T1D2	0.67 b	1.94 c	13.56 a	2.95 a	60.33 a	15.25 a	19.33 a
		T2D1	0.69 b	4.12 b	11.01 b	0.76 b	15.57 b	13.13 b	6.00 a
		T2D2	0.70 b	5.57 b	8.78 c	−0.68 b	−13.95 b	11.44 c	−5.84 a
		T3D1	0.85 ab	11.79 a	4.15 d	−6.91 c	−141.42 c	7.16 d	−97.25 b
		T3D2	0.94 a	13.21 a	2.79 e	−8.32 c	−170.30 c	5.45 e	−154.40 c
	WYJ-24	T1D2	0.64 b	1.63 c	13.37 a	2.89 a	63.95 a	16.23 a	17.85 a
		T2D1	0.65 b	2.76 bc	12.00 b	1.76 ab	38.98 ab	14.35 b	12.34 a
		T2D2	0.67 b	3.90 b	9.44 c	0.62 b	13.64 b	12.04 c	5.08 a
		T3D1	0.80ab	7.49 a	6.84 d	−2.97 c	−65.71 c	10.22 d	−29.52 b
		T3D2	0.88 a	8.61 a	5.70 d	−4.09 c	−90.61 c	9.10 d	−45.58 c
Flowering	HD-5	T1D2	0.67 b	1.94 e	13.64 a	2.95 a	60.34 a	15.19 a	19.42 a
		T2D1	0.69 b	5.07 d	9.09 b	−0.18 b	−3.68 b	11.77 b	−1.46 ab
		T2D2	0.76 b	8.15 c	4.54 c	−3.26 c	−66.76 c	6.51 c	−50.02 b
		T3D1	0.90 a	14.45 b	1.18 d	−9.57 d	−195.85 d	3.30 d	−290.83 c
		T3D2	0.96 a	16.42 a	0.39 d	−11.53 e	−236.08 e	1.21 e	−961.25 d
	WYJ-24	T1D2	0.63 c	1.65 e	13.50 a	2.87 a	63.42 a	15.65 a	18.28 a
		T2D1	0.63 c	3.77 d	10.70 b	0.75 b	16.59 b	13.79 b	5.44 a
		T2D2	0.64 c	6.60 c	6.36 c	−2.09 c	−46.16 c	8.59 c	−24.27 a
		T3D1	0.79 b	12.12 b	1.66 d	−7.60 d	−168.27 d	3.94 d	−193.02 b
		T3D2	0.89 a	13.26 a	0.83 d	−8.74 e	−193.43 e	2.27 e	−387.29 c
Combined	HD-5	T1D2	0.66 c	1.94 e	13.64 a	2.95 a	60.35 a	15.41 a	19.20 a
		T2D1	0.69 bc	4.31 d	9.89 b	0.57 b	11.73 b	12.28 b	4.64 a
		T2D2	0.75 abc	7.84 c	4.95 c	−2.95 c	−60.40 c	6.80 c	−43.42 b
		T3D1	0.91 ab	13.72 b	1.97 d	−8.84 d	−180.91 d	4.44 d	−199.00 c
		T3D2	0.96 a	15.79 a	1.09 e	−10.91 e	−223.26e	2.84 e	−384.96 d
	WYJ-24	T1D2	0.64 b	1.66 e	13.35 a	2.86 a	63.36 a	15.53 a	18.39 a
		T2D1	0.65 b	3.57 d	10.94 b	0.95 b	20.93 b	13.77 b	6.81 a
		T2D2	0.69 b	6.31 c	6.71 c	−1.80 c	−39.76 c	8.73 c	−20.93 a
		T3D1	0.81 a	11.61 b	2.26 d	−7.09 d	−156.93 d	4.28 d	−167.14 b
		T3D2	0.90 a	12.96 a	1.31 d	−8.44 e	−186.80 e	2.79 e	−302.90 c

In the corresponding column for each variety, distinct letters signify statistically significant differences (*p* < 0.05) among the treatments. T denotes the temperature level, and D represents the duration of heat stress. The presented means are derived from three replicates.

**Table 3:** Summary of heat stress treatments conducted in environment-controlled phytotrons.

Year	Site	Cultivar	Timing of Treatment	Temperature Level & Duration
2016	Rugao	HD-5	Booting (BT) (11 August 2016; 12 August 2017) S1 Flowering (FL) (23 August 2016; 26 August 2017) S2 Combined (BT + FL) S3	[T1, T2, T3] × [D1, D2]
		WYJ-24		
2017	Rugao	HD-5	booting (18 August 2016; 24 August 2017) S1 flowering (1 September 2016; 5 September 2017) S2 Combined (BT + FL) S3	
		WYJ-24		

Note: In this context, T refers to temperature levels (Tmax/Tmin), where T1 corresponds to 22 °C/32 °C, T2 to 30 °C/40 °C, and T3 to 34 °C/44 °C. D represents the duration of HS, with D1 indicating 2 days and D2 signifying 4 days.

## Data Availability

Data are contained within the article.
